# Heat Shock Factor 2 Levels Are Associated with the Severity of Ulcerative Colitis

**DOI:** 10.1371/journal.pone.0088822

**Published:** 2014-02-12

**Authors:** Jiarong Miao, Junkun Niu, Kunhua Wang, Yuliang Xiao, Yan Du, Lifeng Zhou, Liping Duan, Shuan Li, Gang Yang, Lifang Chen, Mingxia Tong, Yinglei Miao

**Affiliations:** 1 Department of Gastroenterology, The First Affiliated Hospital of Kunming Medical University, Kunming, P. R. China; 2 Department of General Surgery, The First Affiliated Hospital of Kunming Medical University, Kunming, P. R. China; 3 Department of Clinical Laboratory, The First Affiliated Hospital of Kunming Medical University, Kunming, P. R. China; Massachusetts General Hospital, United States of America

## Abstract

**Background and Aims:**

The morbidity of ulcerative colitis (UC) is increasing in China every year. In addition, there is a lack of accurate diagnostic indices with which to evaluate the activity of the disease. The aim of this study was to identify UC-associated proteins as biomarkers for the diagnosis, and objective assessment of disease activity.

**Methods:**

Differential expression of serum proteins from UC patients compared to normal controls was analyzed by two-dimensional electrophoresis (2-DE) and matrix-assisted laser desorption/ionization-time-of-flight mass spectrometry (MALDI-TOF-MS). The expression of heat shock factor 2(HSF2)in colonic mucosa in Crohn's disease, Behcet's disease, ulcerative colitis, intestinal tuberculosis, infective enteritis, intestinal lymphoma, and normal controls was investigated by immunohistochemistry (IHC). The expression of the HSF2 in colonic mucosa of UC subjects with varying severity of disease was measured by real time-PCR and Western Blots. The expression of HSF2 was inhibited by HSF2 small interfering RNA (siRNA) transfection in Caco-2 cells. The concentrations of HSF2, IL-1β, and TNF-α in serum and IL-1β, and TNF-α in the supernatants of transfected Caco-2 cells were determined by ELISA.

**Results:**

HSF2 was differentially expressed in UC patients compared to normal controls. HSF2 expression was significantly higher in the intestinal mucosa of UC patients compared to other six groups. The results of immunohistochemistry, real time-PCR, Western Blots, and ELISA showed that the expression of HSF2 increased in parallel with the severity of UC. The serum concentration of HSF2 also positively correlated with levels of IL-1β and TNF-α. After down-regulation expression of HSF2 in Caco-2 cells by RNA interference, the productions of IL-1β and TNF-α stimulated by lipopolysaccharide (LPS) increased dramatically.

**Conclusions:**

HSF2 appears to be a potential novel molecular marker for UC activity, and may provide a basis for studies on the pathogenesis and novel therapeutic targets for UC.

## Introduction

Ulcerative colitis (UC) is an inflammatory bowel disease (IBD) characterized by chronic inflammation of the colonic mucosa, often resulting in intermittent bloody diarrhea and abdominal pain [1]. In China, approximately 140,000 cases of UC have been diagnosed during past 15 years, with an 8.5-fold increase during the last 5 years [2]. Although genetic [3], [4], infectious [5], and immunological [6] factors have been postulated to be involved in the pathogenesis of UC, the precise cause of the disease remains unclear. UC is currently diagnosed by clinical, radiologic, endoscopic and histopathological findings. Fecal markers such as calprotectin and lactoferrin have been studied for their ability to identify patients with IBD, assess disease activity, and predict relapse [7]. Serum biomarkers such as C-reactive protein and erythrocyte sedimentation rate have been used to assess inflammatory processes and predict the course of IBD progression [8]. Unfortunately, reliable biomarkers for monitoring disease activity have not been clinically established for use in UC. Therefore, more sensitive and specific biomarkers for UC are needed. Proteomics have been applied to search biomarkers in various diseases [9], [10]. The aim of this study was to analyze protein expression profiles in human serum from patients with UC and normal control subjects using 2-DE and MALDI-TOF MS analysis.

## Materials and Methods

### Patients and samples preparation

Patients were confirmed as UC by endoscopy and pathological examination. All blood samples were drawn under limosis condition at the next morning after diagnosis. All UC patients recruited for this study did not take any medications (or herbal remedies), especially the drugs recommend for UC, such as 5-aminosalicylate, glucocorticosteroid and other immunosuppressive agents. The occurrence of medication was excluded in all patients selected for blood samples collection. Serum and colonic mucosal tissue samples were taken from 40 patients (female to male ratio: 1/1, mean age(years): 36.5, rang from 18–60) with mild to severe UC. Disease activity was assessed using the Mayo Score system from 0 to 12, as described previously [11], in which mild, moderate and severe disease activity were defined by scores of 3–5, 6–10 and 11–12. All samples were collected at the time of diagnosis and stored at −80°C. A sample from each specimen was fixed in formalin for immunohistochemical staining. In addition, serum samples of 40 voluntary healthy controls (female to male ratio: 1/1, mean age(years): 34.5, rang from 18–56) were taken under the same conditions as patients' blood, and colon mucosal tissue samples of 10 people (female to male ratio: 1/1, mean age(years): 36, rang from 22–50) who were suspected to have UC, but then were determined to have Irritable Bowel Syndrome (IBS) instead, were chosen as normal controls. To compare the expression of HSF2 in UC with other diseases, paraffin-embedded intestinal tissue samples on-file at the Department of Pathology were obtained: intestinal tuberculosis (31 cases), Crohn's disease (29 cases), intestinal lymphoma (32 cases), Behcet's disease (28 cases), infective enteritis (32 cases). Written informed consent was obtained from all patients at the time samples were taken, and the study was approved by the Ethics Committee of The First Affiliated Hospital of Kunming Medical University, Kunming, China.

### Two-dimensional electrophoresis (2-DE)

Fasting blood, 5 ml, was drawn from the patients, and centrifuged at 2,000 rpm for 20 min. Non-hemolyzed serum was collected, and 300 µl from 10 cases were mixed. Highly abundant albumin and IgG were removed with an albumin/IgG removal kit (Calbiochem, CA, USA) according to the manufacturer's instructions. Total protein concentrations of serum samples were determined by a Dye Reagent protein assay kit (Bio-Rad, CA, USA). Aliquots were kept at −80°C for two dimensional gel electrophoresis which was performed using an Ettan IP Gphor Isoelectric Focusing System according to the method of Boguth et al [12]. In brief, samples containing about 250 µg of treated serum proteins were dissolved in rehydration buffer, and isoelectrically focused at 250 V for 30 min, 1000 V for 2 h, 10 000 V for 5 h, and 10 000 V for a total of 60 000 Vh/gel. After isoelectric focusing (IEF), each immobilized pH gradient (IPG) strip was soaked in equilibration solution containing dithiothreitol (DTT) and iodoacetamide, and then placed on in contact with the top surface of SDS-PAGE and sealed with agarose. Separation in the second dimension gel was performed using a Tris-glycine running buffer, at a power setting of 5 mA/gel for 0.5 h and 30 mA/gel for 2 h, thereafter at a temperature of 17°C. The protein spots were visualized by silver staining and scanned on Umax Powerlook 1100 scanner. Target gels were analyzed with PDQuest 7.1.0 software including spot detection, background subtraction, and matching. Each assay was replicated three times, and protein spots for comparative analyses were detected on all the gels. Only the spots present in all three replicate gels and qualitatively consistent in size and shape in the replicate gels were considered.

### In-gel Digestion

Spots from 2-DE were excised and digested with modified trypsin (Roche, Mannheim, Germany) as previously described by Millares et al [13]. In brief, gel particles were washed and dehydrated with acetonitrile. Proteins were reduced with DTT, and S-alkylated with iodoacetamide. Gel particles were washed with NH_4_HCO_3_ and then dried under vacuum rehydrated with the digestion solution. After incubation for 30 min at 4°C, supernatants were replaced by NH_4_HCO_3_, and gel particles were incubated overnight at 37°C. Trifluoroacetic acid, 2%, was added to end the digestion.

### MALDI-TOF MS analysis for protein identification

Samples were mixed (1∶1) with a saturated matrix solution (α-cyano-4-hydroxycinnamic acid prepared in 60% acetonitrile/0.1% trifluoroacetic acid). Matrix-assisted laser desorption ionization time of flight (MALDI-TOF) mass spectra were recorded on a BIFLEXIV mass spectrometer [14]. All spectra were collected in a positive ion reflector mode with a delayed extraction mass accuracy of about 100 ppm. The specific parameters were as follows: the ion acceleration voltage 19 kV, and nitrogen laser operating at a wavelength 337 nm. MS spectra were obtained in the mass range between 2000 and 3000 Da. The singly charged peaks were analyzed using an interpretation method present in instrument software. The nine most intense peaks were selected, and MS/MS spectra were generated automatically, excluding those from the matrix, due to trypsin autolysis peaks. Spectra were processed and analyzed by the Matrix science web which uses internal Mascot 2.1 software to search for peptide mass fingerprints and MS/MS data. Searches were performed against the SWISS-PROT and NCBI non-redundant (nr) protein database.

### Immunohistochemistry (IHC) analysis

Colonic mucosal tissues were cut into three sections (5 µm each) with freezing microtome, and put them on the same slide. After fixation, we performed immunohistochemical staining of HSF2 in all cohorts. The HSF2 (1∶50, Santa Cruz, TX, USA) primary antibodies were used for antigen detection. HRP-Polymer anti-mouse/rabbit IHC Kit and DAB (3, 3′-diaminobenzidine) substrate kit were used as detecting reagents according to the manufacturer's recommendations (Maxim Biotech, Fuzhou, CHN). The slides were counterstained with hematoxylin, fixed by Scott's solution, and dehydrated with various concentrations of ethanol.

Slides were mounted with permount mounting medium and observed under a light microscope, randomly selecting four visual fields on each slide.

Semi-quantitative analysis of staining intensity for the HSF2 protein was performed using the HPIAS-2000 image analysis software (Tongji Qianping Imaging Inc., Wuhan, China) according to Chen [15] and Wang et al. [16]. In brief, four highly magnified visual fields (10×40 magnification) (with no overlap) from each tissue section were randomly selected, and theirs digitalized images were submitted to the image analysis software. HSF2 positive cells defined as those having brown-yellow granules in the cytoplasm and/or nucleus. Integral optical density was automatically measured by the computer with image analysis software. The mean optical density represented the relative expression levels of HSF2 protein. Assessments were performed by two independent pathologists from The First Affiliated Hospital of Kunming Medical University, who were unaware of the HSF2 status.

### Quantitative real-time PCR

Total RNA from the colonic mucosal tissue was extracted with TRIzol reagent (Invitrogen, CA, USA) according to the manufacturer's protocol. The first-strand cDNA synthesis was performed with a cDNA synthesis kit (TaKaRa, Dalian, China) according to the manufacturer's instructions. Quantitative real-time PCR was carried out using an SYBR Green real-time PCR kit (TaKaRa, Dalian, China) under the following conditions: initial denaturation at 95°C for 1 min, followed by 40 cycles at 95°C for 15 s, 60°C for 15 s, and 72°C for 20 s. The primer sequences were as follows: human HSF2 (139 bp): 5′-ATAAGTAGTGCTCAGAAGGTTCAGA-3′ (forward) and 5′-GAATAACTTGTTGCTGTTGTGCATG-3′ (reverse), GAPDH (107 bp): 5′-ATGGGGAAGGTGAAGGTCG-3′ (forward) and 5′-GGGGTCATTGATGGCAACAATA-3′ (reverse). Each sample was run three times. The data from the real-time PCR were analyzed with the delta–delta Ct method and normalized to the amount of GAPDH cDNA as an endogenous control.

### Western blotting

Colonic mucosal tissue samples and Caco-2 cells were homogenized in immunoprecipitation assay buffer (Roche, Mannheim, Germany) with a protease inhibitor cocktail (Roche, Mannheim, Germany). Homogenates were centrifuged at 4°C, 12000 rpm for 10 min, and the supernatants were collected to determine protein concentration using the Dye Reagent protein assay kit (Bio-Rad, CA, USA). Samples containing 50 µg of protein were loaded on an SDS-polyacrylamide gel electrophoresis, and then electrotransferred onto a PVDF membrane (Millipore, MA, USA). The membrane was blocked with 3% BSA and then incubated with antibodies specific for HSF2 (1∶1000, Santa Cruz, TX, USA), Monoclonal ANTI-FLAG M2 antibody (1∶5000, Sigma Aldrich, MO, USA), and β-actin (1∶5000, Santa Cruz, TX, USA ) overnight at 4°C. Membranes were incubated with appropriate peroxidase-conjugated secondary antibodies (Santa Cruz, TX, USA). The blots were visualized using Super Signal West Pico reagent (Pierce, IL, USA) and the chemiluminescence signal was captured using a ChemiDoc XRS system (Bio-Rad, CA, USA).

### Cell culture

Caco-2 cells, a human colon adenocarcinoma cell line that displays enterocyte-like features in culture, were obtained from Cell Bank of Type of Culture Collection of Kunming Institue of Zoology, Chinese Academy of Sciences. Cells were grown at 37°C in 5% CO_2_ in Dulbecco's modified Eagle's medium (DMEM, HyClone, NY, USA) supplemented with 10% fetal bovine serum. Cells, between passages 5 and 25, were seeded at a density of 100,000 cells/ml onto 12-well tissue culture plates (Corning, NY, USA) and used at 40%–50%, 70%–80% confluence for RNA interference and plasmid transfection, respectively. After overnight transfection, the Caco-2 cells were treated with 50 ng/ml LPS (Sigma Aldrich, MO, USA) or equal physiological saline (as a normal cotrol) for 18 h, and the culture supernates were harvested for cytokine assays.

### RNA interference and transfection

The sequences of HSF2 siRNA (Cat.No:SI04210598, Qiagen, Frankfurt, GER) were as follow: Target sequence:5′-CTGCGCCGCGTTAACAATGAA-3′; Sense strand: 5′GCGCCGCGUUAACAAUGAATT-3′; Antisense strand: 5′-UUCAUUGUUAACGCGGCGCAG-3′. A recombinant plasmid encoding HSF2 gene and containing an amino-terminal FLAG sequence (DYKDDDD), HSF2-FLAG, has been constructed as described by Chubet et al [17]. HSF2 siRNA and HSF2-FLAG recombinant plasmid were transfected into Caco-2 cells by Lipofectamine means according to manufacturer's protocols (Invitrogen, CA, USA).

### ELISA assay

The concentrations of HSF2, IL-1β, and TNF-α in serum and IL-1β, and TNF-α in the supernatants of transfected Caco-2 cells were determined by ELISA kits according to manufacturer's protocols (HSF2, CUSABIO, Wuhan, China; IL-1β and TNF-α ELISA kits, Neobioscience, Beijing, China, respectively).

### Statistical analysis

All data were presented as means ± standard deviation (SD). Statistical analyses were performed using SPSS17.0 statistical software. Multi-group data analyses were tested by one-way analysis of variance (ANOVA). Correlation analyses between the variables were assessed using the Pearson Test which gives a correlation coefficient (Pearson “r”) and a “*p*” value. A *p* value of <0.05 was considered as the minimum level of significance in all cases.

## Results

### Identification of UC-associated proteins by MALDI-TOF MS

**Figure 1:**
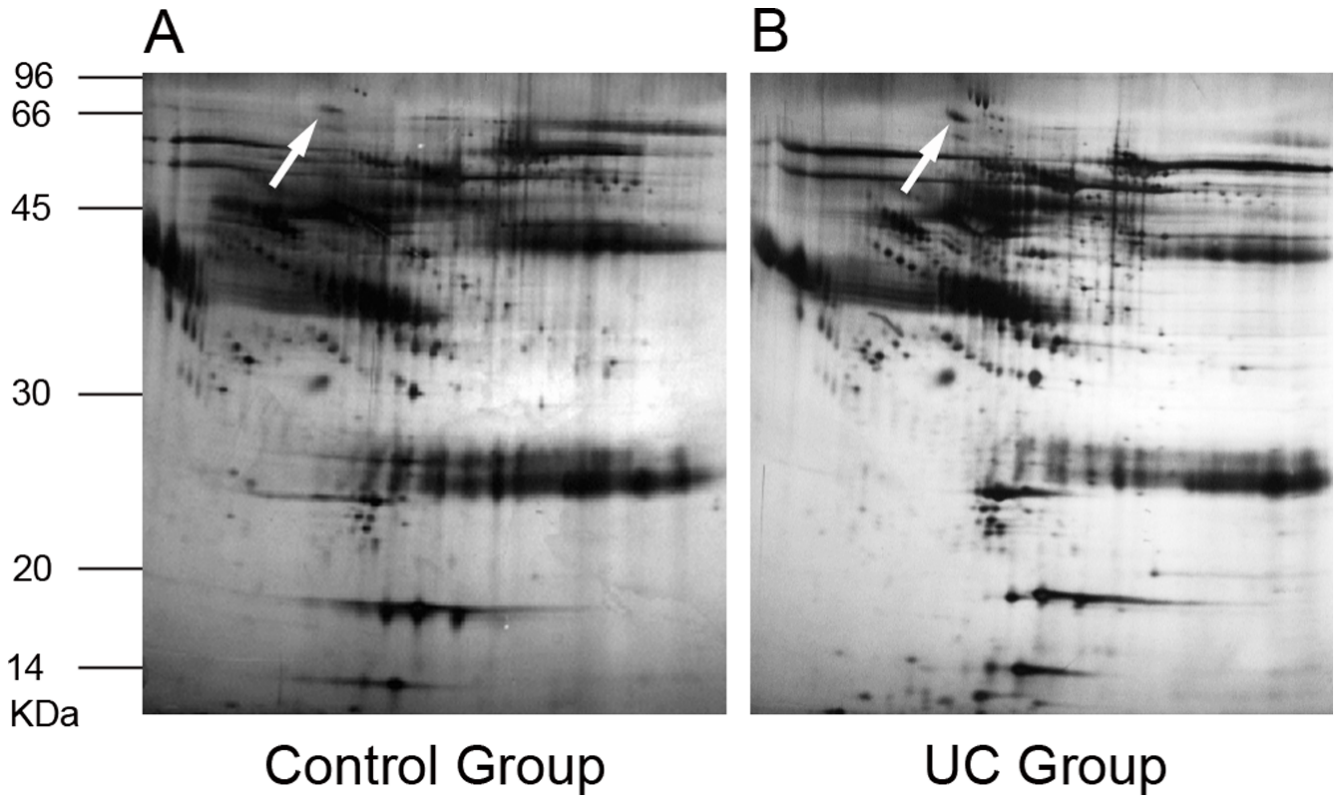
Total proteins from serum of normal control subjects and UC patients were analyzed by 2-DE. The proteins were separated by IEF (pH 3–10) and 10% SDS-PAGE and subsequently processed with silver staining. The spot marked with white arrow represented HSF2. **A**: Total proteins from serum of normal controls that were analyzed by 2-DE. **B**: Total proteins from serum of UC patients that were analyzed by 2-DE.

As shown in Figure 1, a total of 39 protein spots in the UC groups with differential expression levels were found on 2-DE. Of the 39 protein spots, 12 proteins were eventually identified by MALDI-TOF-MS, and 9 pieces of peptide mass fingerprinting (PMF) were obtained (Table 1). Among these identified proteins, 6 proteins were over-expressed: haptoglobin, aldehyde reductase, receptor tyrosine kinase, pericentriole material 1, heat shock factor 2 and apolipoprotein C-III. Three proteins were under-expressed in sera of patients with UC: tropomyosin 3, filamin A interacting protein 1 and keratin1. To clearly demonstrate the difference in expression of serum proteins between UC patients and normal controls, representative protein spots were picked up from 2-DE (Figure 1). These representative proteins had much higher expression in serum of patients with UC than that in normal controls.

**Table:** **Table1.** Protein spot identification by MALDI-TOF MS in conjunction with PMF with SWISS-PROT and NCBI non-redundant protein database searching.

Protein Total Spot Score	Protein Identified	Accession Number	Protein Score
Spot1 (181)	Haptoglobin	gi|3337390	208
Spot2 (43)	Aldehyde reductase	gi|225939	60
Spot3 (53)	Receptor tyrosine kinase	gi|225939	62
Spot4 (45)	Pericentriole material 1	gi|450277	55
Spot5 (50)	Heat shock factor 2	gi|13529107	66
Spot6 (39)	Tropomyosin 3	gi|55665776	56
Spot7 (49)	Filamin A interacting protein 1	gi|31542634	62
Spot8 (83)	Apolipoprotein C-III	IPI00657670	97
Spot9 (56)	Keratin 1	IPI00220327	114

Nine protein spots were excised from the 250 mg protein-loaded gel and analyzed by MALDI-TOF MS. A local MASCOT PMF search was performed with its MOWSE-based score (significance threshold score >51, P<0.05). Each EST sequence hit was submitted to a BLASTp search against the entire NCBI nr protein database. The accession number and protein name of nine protein spots are listed.

### Immunohistochemical staining of HSF 2 in colonic mucosa of UC

**Figure 2:**
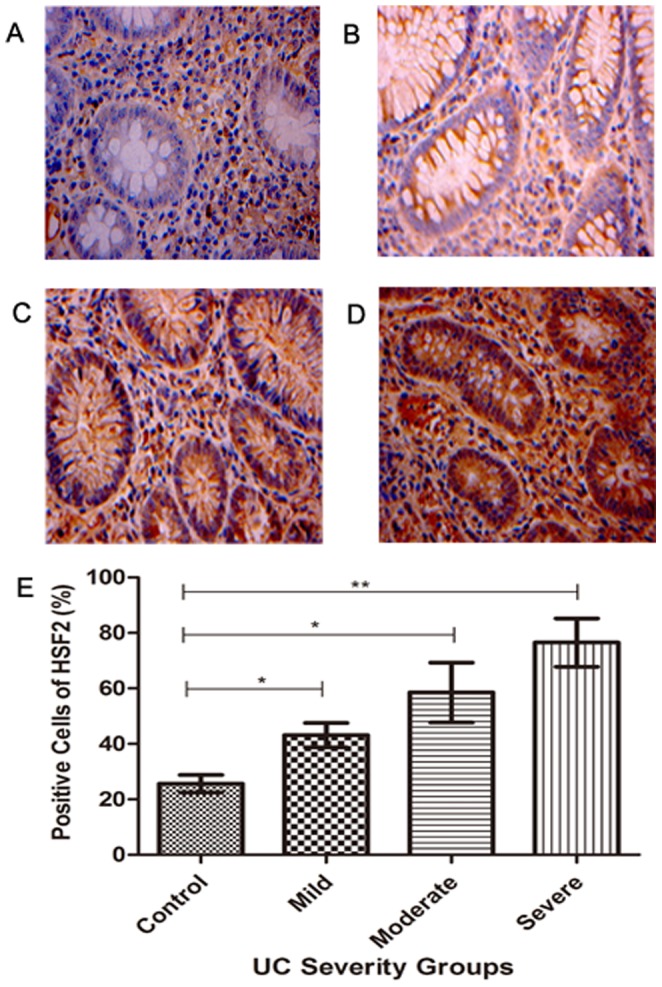
Expression of HSF2 in colonic mucosa tissues of normal control and UC was examined by IHC. **A**: Representative normal colonic mucosa tissue was negatively stained with anti-HSF2 antibody; **B**, **C** and **D**: Representative UC colonic mucosal tissues of mild, moderate and severe group were positively stained with anti-HSF2 antibody, respectively (magnification, ×400); **E**: Positive cell expression of HSF2 was compared. All IHC results were read automatic image analyzer, measured by HPIAS-2000 analytic software. *, p<0.05; **, p<0.01.

The expression profiles of HSF2 in colonic mucosa were examined by IHC (Figure 2). HSF2 was expressed in stromal cells and almost undetectable on the epithelial cells in normal intestinal mucosa (Figure 2A), but widely expressed in intestinal epithelial cells and stromal cells in UC group (Figure 2B, C and D), The expression level of HSF2 in mucosal tissues from the group with severe disease was the highest of these three groups (p<0.01), and HSF2 expression in the moderate group was higher than that in mild group(p<0.05). The expression of HSF2 increased significantly with increasing severity of disease.

### Immunohistochemical staining of HSF 2 in colonic mucosa of ulcerative colitis, Crohn's disease, Behcet's disease, intestinal tuberculosis, infective enteritis and intestinal lymphoma and normal controls

**Figure 3:**
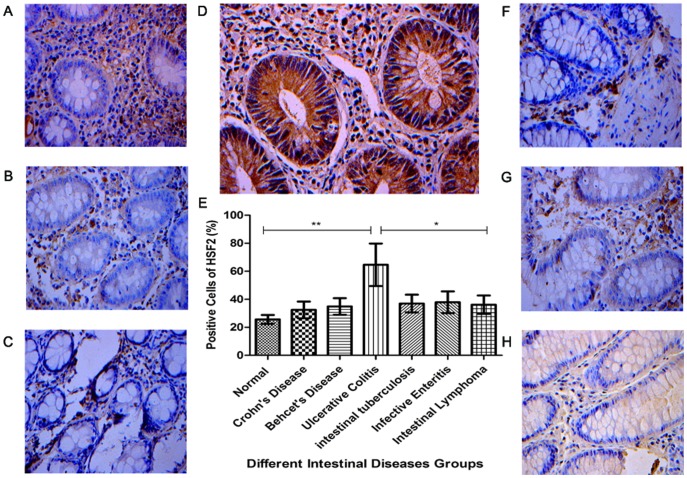
Expression of HSF2 in colonic mucosa tissues was examined by IHC. **A**: Representative normal colonic mucosa tissue was stained with anti-HSF2 antibody; B, **C**, **D**, **F**, **G** and **H**: Representative colonic mucosa tissues of Crohn's disease, Behcet's disease, ulcerative colitis, intestinal tuberculosis, infective enteritis, and intestinal lymphoma were stained with anti-HSF2 antibody, respectively (magnification, ×400); **E**: Positive cell expression of HSF2 was compared. All IHC results were read automatic image analyzer, measured by HPIAS-2000 analytic software. *, p<0.05;**, p<0.01.

The results of IHC indicated that the expression of HSF2 in the intestinal mucosa of UC patients was significantly higher than that in other six groups (Figure 3), (p<0.01, for normal controls, Crohn's disease, Behcet's disease), (p<0.05 for intestinal tuberculosis, infective enteritis and intestinal lymphoma). However, there was no significant difference among six groups (p>0.05).

### Transcription and expression of HSF2 in colonic mucosa of UC

**Figure 4:**
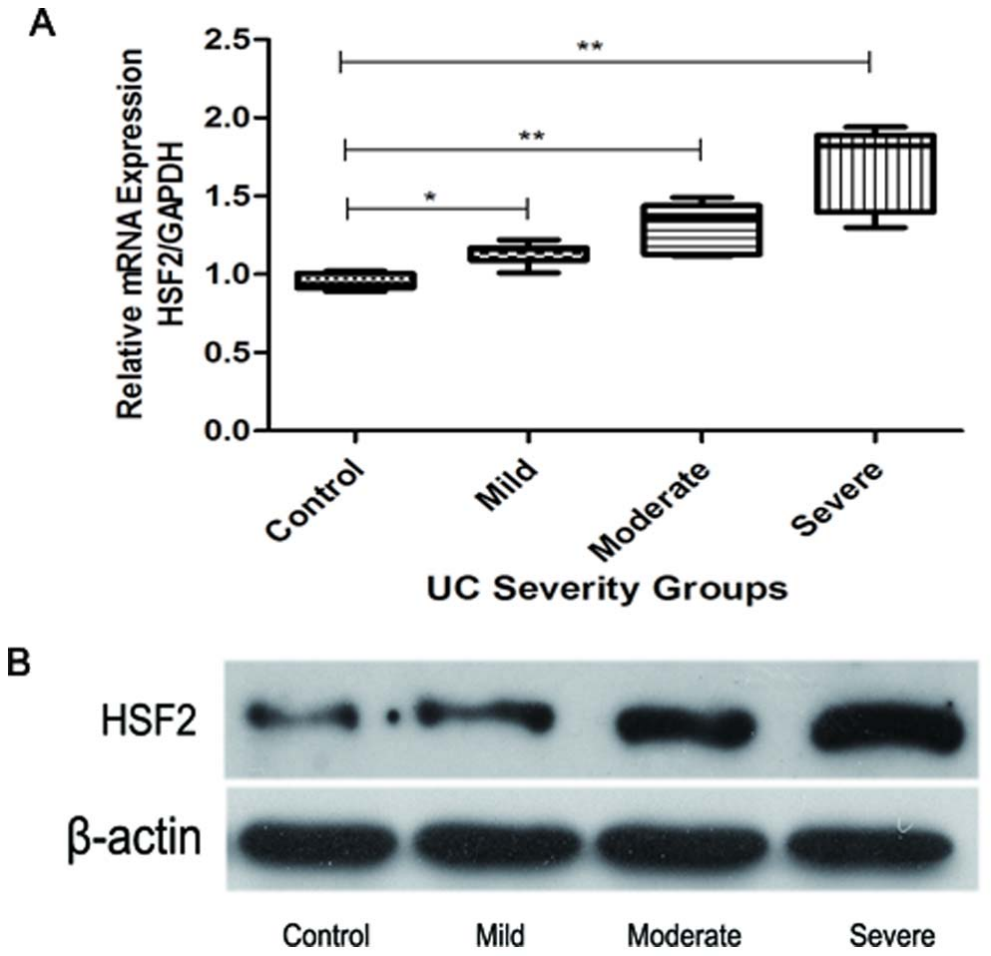
Expression of HSF2 in colonic mucosa tissues of UC was examined by real- time PCR and Western Blotting. **A**: the mRNA transcriptional levels of UC groups of varying severity. The data from the real-time PCR were analyzed with the delta–delta Ct method and normalized to the amount of GAPDH cDNA as an endogenous control. B: the protein expression levels of different UC severity groups, β-actin as an endogenous control. *, p<0.05; **, p<0.01.

Real time-PCR results (Figure 4A) showed that the mRNA transcriptional level of HSF2 in colonic mucosa increased with disease severity. The mRNA levels of HSF2 in mucosal tissues from the severe group was the highest of the three groups (p<0.01), and that of the moderate group was higher than that in mild group and normal controls (p<0.01, for all). In addition, Western Blot results (Figure 4B) showed that the protein levels of HSF2 in colonic mucosa increased with increasing disease severity. There were significant differences among different UC severity groups.

### Concentrations of HSF2, IL-1β, and TNF-α

**Figure 5:**
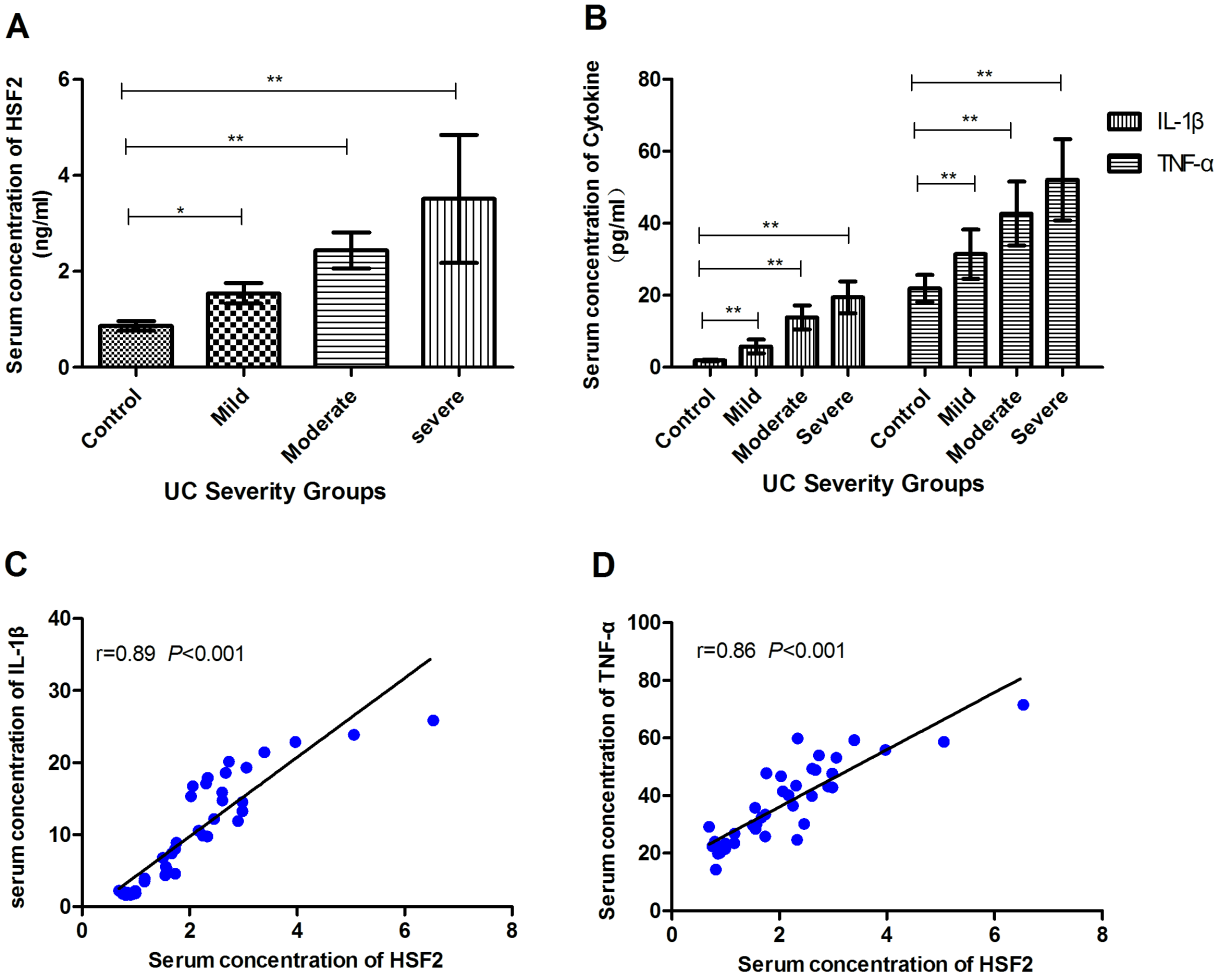
The concentration of HSF2, IL-1β and TNF-α in serum was determined by ELISA. **A**: serum concentration of HSF2 in patients with UC of varying severity. B: serum concentrations of IL-1β and TNF-α of different UC severity groups. C: Correlation analysis between the concentrations of HSF2 and IL-1β. D: Correlation analysis between the concentrations of HSF2 and TNF-α.Correlation analysis was assessed using the Pearson Test which gives a correlation coefficient (Pearson “r”) and a “*p*” value. *, p<0.05; **, p<0.01.

As shown in Figure 5, the serum concentrations of HSF2, IL-1β and TNF-α increased with disease severity. In addition, the serum concentrations of HSF2 were positively correlated with IL-1β (r = 0.89, *p*<0.001), and with TNF-α (r = 0.86, *p*<0.001).

**Figure 6:**
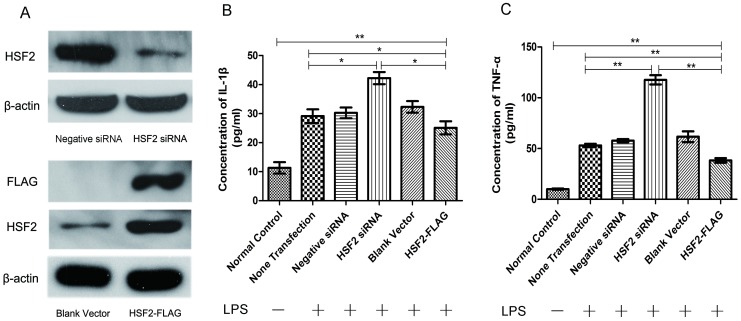
The secretion of IL-1β and TNF-α in Caco-2 cells after enhanced expression or siRNA of HSF2. A: The HSF2 expression levels after HSF2 RNA interferenc and HSF2-FLAG recombinant plasmid transfection, β-actin as an endogenous control. B: The productions of IL-1β in Caco-2 cells supernates stimulated or not by LPS (50 ng/ml). C: The productions of TNF-α in Caco-2 cells supernates stimulated or not by LPS (50 ng/ml). *, p<0.05;**, p<0.01.

After down-regulation expression of HSF2 in Caco-2 cells by RNA interference (Figure 6A), the productions of IL-1β (Figure 6B) and TNF-α (Figure 6C) stimulated by LPS increased dramatically compared to the other four groups (p<0.01). Enhanced expression of HSF2 by plasmid transfection (Figure 6A) resulted in significantly decresased production of these two cytokines (Figure 6B and C) compared to other LPS-stimulated cell groups (p<0.05).

## Discussion

Because of the dearth of molecular markers for UC, colonoscopy with colonic mucosal biopsy is currently routine for diagnostic evaluation for UC. Biomarkers have been considered to be objective, and non-invasive measurements of disease activity.

Alteration in the levels of some serum proteins have been shown to be early signs of altered physiology and may be indicative of disease [18]. In the present study, we identified 12 differential protein spots using MALDI-TOF-MS, and obtained nine pieces of PMF. PMFs were identified through searches of the SWISS-PROT and NCBI nr databases. Among these identified proteins, six (heat shock factor protein 2, haptoglobin, apolipoprotein C-III, receptor tyrosine kinase, aldehyde reductase and pericentriolar material 1) were found to be up-regulated, and three (keratin 1, filamin A-interacting protein 1 and tropomyosin 3) were found to be down-regulated.

Over the last years evidence has accumulated that HSF1and HSPs are very important for the repair of colonic mucosa epithelium in inflammatory bowel disease and they suppress proinflammatory genes relevant to its pathogenesis [19]–[22], but little is known about the function of HSF2 in the pathogenesis of UC, most studies on HSF2 have been on protein misfolding diseases, delaying aging, development of embryo and sperm [23]–[25].

Based on the background facts and the findings of proteomic analysis, an up-regulated protein HSF2 was selected for further validation in the progression of UC. UC begins in the rectum and spreads variably to the proximal colon [26], and is characterized by continuous lesion, crypt abscess and abnormal branching [27]. Crypt abscesses are early lesions observed in inflammatory bowel disease, particularly in UC [28]. To some extent, crypt cells serves as a protective barrier between noxious stimuli and the sterile host environment. Exposure to such noxious stimuli may result in increased proliferation of crypt cells, secretion of enzymes, inflammatory cytokines and HSPs [29]. The results of the current study showed that the expression of HSF2 was up-regulated in the serum and intestinal mucosa of UC patients, suggesting that multiple stimuli might cause human stress responses, and high expression of HSF2 improves stress response. The expression of HSF2 in the intestinal mucosa of UC patients was significantly higher than that in normal controls, suggesting that HSF2 may be involved in the repair of colonic mucosa epithelium through activation of some protection proteins in response to intestinal mucosa membrane damage.

Recently, HSF1 has been shown to inhibit the expression of proinflammatory cytokines such as TNF-α and IL-1β by regulating the expression of the HSP, and suppressing key transcription factors of inflammatory signaling pathways, such as NF-κB and AP-1 [30]. The current data showed that serum concentrations of HSF2 were positively correlated with two proinflammatory factors, TNF-α and IL-1β. After down-regulation expression of HSF2 in Caco-2 cells by RNA interference, the secretions of these two cytokines stimulated by LPS increased dramatically, while enhanced expression of HSF2 by plasmid transfection resulted in significantly decresased production, suggesting that HSF2 might directly or indirectly affect inflammation-related transcription factors and down-regulates inflammatory cytokines to overcome inflammation.

It is important to understand the pathogenesis of UC and identify specific biomarkers and biological therapeutic targets [31], [32]. Our results showed that HSF2 was over expressed in UC, and the increases paralleled the severity of disease. This suggests that HSF2 might be an endogenous protective factor against UC. This study will enable HSF2 as a potential novel molecular marker for UC and provide the basis for novel biological therapeutic targets.
